# Engaging patients and families to create a feasible clinical trial integrating palliative and heart failure care: results of the ENABLE CHF-PC pilot clinical trial

**DOI:** 10.1186/s12904-017-0226-8

**Published:** 2017-08-31

**Authors:** Marie Bakitas, J. Nicholas Dionne-Odom, Salpy V. Pamboukian, Jose Tallaj, Elizabeth Kvale, Keith M. Swetz, Jennifer Frost, Rachel Wells, Andres Azuero, Konda Keebler, Imatullah Akyar, Deborah Ejem, Karen Steinhauser, Tasha Smith, Raegan Durant, Alan T. Kono

**Affiliations:** 1 School of Nursing and Department of Medicine, Division of Gerontology, Geriatrics, and Palliative Care, University of Alabama at Birmingham, Birmingham, AL USA; 2 School of Nursing, University of Alabama at Birmingham, 1720 2nd Ave South, MT 412C, Birmingham, AL 35294 USA; 3 Department of Medicine, Division of Cardiovascular Diseases, University of Alabama at Birmingham, Birmingham, AL USA; 4 Department of Medicine, Division of Gerontology, Geriatrics and Palliative Care, University of Alabama at Birmingham, Birmingham, AL USA; 5 Cardiology, Dartmouth-Hitchcock Medical Center/Geisel School of Medicine at Dartmouth, Lebanon, NH USA; 6 School of Nursing, University of Alabama at Birmingham, Birmingham, AL USA; 7 Faculty of Health Sciences, Nursing Department, Hacettepe University, Ankara, Turkey; 8 Center for Health Services Research in Primary Care, Durham VA Medical Center, Durham, NC, Division of General Internal Medicine, Department of Medicine, Duke University, Durham, NC USA; 9 Department of Medicine, Division of Preventative Medicine, University of Alabama at Birmingham, Birmingham, AL USA

**Keywords:** Heart failure, Palliative care, Caregiver, Telehealth, Rural, Intervention development

## Abstract

**Background:**

Early palliative care (EPC) is recommended but rarely integrated with advanced heart failure (HF) care. We engaged patients and family caregivers to study the feasibility and site differences in a two-site EPC trial, ENABLE CHF-PC (**E**ducate, **N**urture, **A**dvise, **B**efore **L**ife **E**nds **C**omprehensive **H**eartcare for **P**atients and **C**aregivers).

**Methods:**

We conducted an EPC feasibility study (4/1/14–8/31/15) for patients with NYHA Class III/IV HF and their caregivers in academic medical centers in the northeast and southeast U.S. The EPC intervention comprised: 1) an in-person outpatient palliative care consultation; and 2) telephonic nurse coach sessions and monthly calls. We collected patient- and caregiver-reported outcomes of quality of life (QOL), symptom, health, anxiety, and depression at baseline, 12- and 24-weeks. We used linear mixed-models to assess baseline to week 24 longitudinal changes.

**Results:**

We enrolled 61 patients and 48 caregivers; between-site demographic differences included age, race, religion, marital, and work status. Most patients (69%) and caregivers (79%) completed all intervention sessions; however, we noted large between-site differences in measurement completion (38% southeast vs. 72% northeast). Patients experienced moderate effect size improvements in QOL, symptoms, physical, and mental health; caregivers experienced moderate effect size improvements in QOL, depression, mental health, and burden. Small-to-moderate effect size improvements were noted in patients’ hospital and ICU days and emergency visits.

**Conclusions:**

Between-site demographic, attrition, and participant-reported outcomes highlight the importance of intervention pilot-testing in culturally diverse populations. Observations from this pilot feasibility trial allowed us to refine the methodology of an in-progress, full-scale randomized clinical efficacy trial.

**Trial registration:**

Clinicaltrials.gov NCT03177447 (retrospectively registered, June 2017).

**Electronic supplementary material:**

The online version of this article (doi:10.1186/s12904-017-0226-8) contains supplementary material, which is available to authorized users.

## Background

Of the 6 million U.S. individuals with heart failure (HF), approximately 300,000 will die each year. By 2030, HF prevalence is expected to swell by 46% to over 8 million [1]. Evidence-based advances in medication management (e.g. ACEIs, beta-blockers), coronary revascularization, mechanical circulatory support devices, and cardiac resynchronization have lengthened HF survival [2, 3]. However, these added years of life are often accompanied by significant morbidity and prognostic uncertainty requiring difficult discussions and decisions about treatments and quality of life (QOL) [2, 4, 5]. These burdens often extend beyond the individual with HF to family members who must take on new roles to assist patients with disease management [6]. These caregivers can develop needs [7] that, when unmet, lead to psychological stress and poorer health [8, 9].

Recent professional guidelines recommend involvement of palliative care for New York Heart Association (NYHA) Class III/IV and American Heart Association (AHA) Stage C/D HF patients undergoing advanced therapies, facing difficult medical decisions, having complex or refractory symptoms, and having overstrained caregivers [10–15]. Yet only 1-out-of-3 HF patients receive palliative care and usually not until the final weeks to days before death [16]. Therefore, integrating palliative care earlier in the HF trajectory, when patients are relatively healthy and functional, may help patients and their families cope and live better with advanced disease [17–20].

High quality trials in cancer have demonstrated positive patient and family caregiver outcomes from early palliative care (EPC) [21, 22]. However, given the difficulties in prognostication, the prevalence of sudden cardiac death, [23] and an erratic illness trajectory, [24] it is not clear when or how to integrate palliative care in HF [25]. Furthermore, trials of EPC have rarely included persons with low income and education, of a minority race, and who reside in rural, medically-underserved areas [25–29]. Thus, it is imperative to develop models of EPC that are responsive to the HF trajectory, and also are tailored to be culturally appropriate for minority and underserved populations for whom HF can have particularly pernicious effects.

To address these challenges, we actively engaged patients and family members with diverse socioeconomic and racial backgrounds to aid in further refining and culturally-tailoring ENABLE CHF-PC (**E**ducate, **N**urture, **A**dvise, **B**efore **L**ife **E**nds **C**omprehensive **H**eartcare for **P**atients and **C**aregivers), a telephonic EPC intervention for rural-dwelling, underserved HF patients and their family caregivers. In a proof-of-concept, formative evaluation study [30], we translated materials and protocols from our successful EPC ENABLE oncology model [31–33] to a HF population. This study demonstrated acceptability, feasibility, and a signal of potential efficacy in an educationally, socioeconomically, and racially homogeneous sample of 11 patient-caregiver dyads [30] Thus, the current ENABLE CHF-PC feasibility trial was expanded to include an additional site in the southeastern U.S. that had greater racial and cultural diversity in order to identify intervention acceptability and feasibility and thus greater generalizability to a broader U.S. population. The purpose of this study was to: 1) determine the feasibility of recruiting and retaining a rural, racially-diverse sample of patient-caregiver dyads for 24 weeks and 2) explore longitudinal patient and caregiver outcomes including QOL, global health, anxiety, and depression to inform intervention measures and the need for additional protocol modifications for a larger clinical efficacy trial.

## Methods

### Study design

In this feasibility study, conducted April 1, 2014 to December 31, 2015 individuals with AHA Stage C/D and/or NYHA Class III/IV HF and their family caregivers received the ENABLE CHF-PC intervention and were followed for 24 weeks. The study protocol was approved by the institutional review boards of Dartmouth College (Lebanon, New Hampshire) and the University of Alabama at Birmingham (Birmingham, Alabama) and all participants provided written informed consent.

### Setting and sample

Study participants were recruited from cardiology clinics at 1) Dartmouth-Hitchcock Medical Center (DHMC), Lebanon, NH, which serves a largely rural, white population in a state ranked lowest in religiosity, and 2) the University of Alabama at Birmingham (UAB), Birmingham, AL, which serves a diverse rural-urban population that includes a large proportion of Blacks/African-Americans in a state ranked highest in religiosity [34]. Study coordinators at both sites reviewed outpatient cardiology clinic schedules to identify eligible patients. Following physician approval, a study coordinator approached patients and their caregivers during a clinic appointment to explain the study and obtain consent.

Patient inclusion criteria were: 1) diagnosis of NYHA Class III/IV and/or AHA Stage C/D HF; 2) English speaking; 3) ≥50 years of age; and 4) completion of baseline questionnaires. Exclusion criteria were: 1) dementia or impaired cognition (Callahan score ≤ 4) [35], 2) active Axis I psychiatric or substance use disorder; and 3) non-correctable hearing impairment. Patients were asked to nominate a caregiver for participation, defined as “someone who knows you well and is involved in and has knowledge of your medical care.” Caregivers were only excluded for non-correctable hearing loss.

### The ENABLE CHF-PC intervention

**Fig. 1:**
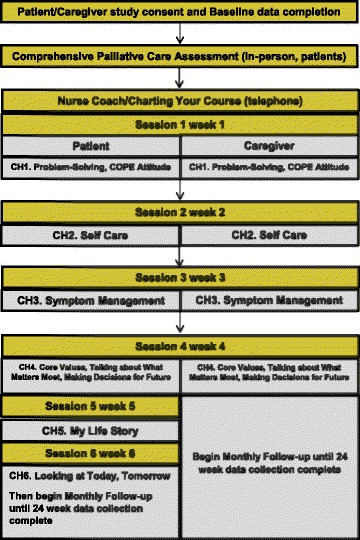
Study Schema

A comprehensive description of the evolution and development of ENABLE CHF-PC has been described in detail elsewhere [30]. Briefly, the ENABLE CHF-PC intervention (Fig. 1) tested in this study included: 1) an in-person outpatient palliative care consultation (caregiver invited to attend) following National Consensus Guidelines [36], 2) weekly, semi-structured palliative care nurse coach (patients: 6 sessions; caregivers: 4 sessions) telephone and monthly follow-up sessions using *Charting Your Course*, an educational guidebook. Sessions, conducted weekly, covered the following topics problem solving, self-care, symptom management, decision-making and advance care planning, and life review (patients only) that were tailored to individual participant needs. The life review sessions were based on Steinhauser and colleagues’ Outlook intervention [37]. The goal of the sessions was to encourage participants to feel empowered and to develop skills that would assist them to make value-driven decisions about their medical and life-sustaining treatment choices as their disease worsened: Patients and caregivers were assigned separate nurse coaches to increase their sense of confidentiality.

Five nurse coaches each received 20 h of training including self-study of intervention protocols and scripts and interactive role-play of 10 digitally-recorded practice sessions. The nurse coaches were debriefed on their training sessions by the principal investigator (PI) (MB) and co-investigator (co-I) (JND-O) and were provided with constructive feedback. Intervention fidelity was maintained through standardized training, the use of structured interventionist scripts, use of standardized session documentation templates, and weekly PI and co-I supervisory team meetings [38].

### Data collection and measures

Study coordinators completed measures with patients and caregivers by phone at baseline, 12- and 24-weeks. Participants received a $10 check for each completed measurement occasion. Baseline demographics included age, gender, race/ethnicity, religion, marital and work status, educational level, and medical insurance. Clinical characteristics abstracted from electronic health records included NYHA class, ejection fraction, presence of an implanted heart device, medications, and laboratory data. These data were entered into the Seattle Heart Failure Model (SHFM) web-based calculator to compute 1, 2 and 5-year survival estimates (https://depts.washington.edu/shfm/). Nurse coaches also informed patients and caregivers that the purpose of this pilot trial was to determine intervention and study procedure acceptability in a new patient population (those with heart failure from diverse socioeconomic cultures). Hence the nurse coach would be actively seeking their critique and feedback throughout the intervention in order to make improvements for future patients. Nurses recorded sessions, and actively tracked patient and caregiver feedback on intervention components that were found to be helpful or in need of improvement in a Research Electronic Data Capture (REDCap) database [39]. Additional file 1: Table S1 shows patient- and caregiver-reported outcome measures.

### Statistical analysis

The feasibility primary aim was determined by monitoring participants’ study status (enrolled, deceased, lost to follow-up) and calculating intervention and measurement completion rates (e.g. actual # completed/possible # per protocol). Patient and caregiver demographic characteristics were tabulated and compared between sites with bivariate tests of association and effect sizes (Cohen’s *d* [40] or *d*-equivalent [41] or nominal variables). We assessed associations between baseline characteristics and participant attrition using simple logistic regressions. We used estimated odds ratios to determine associations between patient characteristics and attrition.

We used longitudinal, fitted, linear mixed methods, adjusted for covariates associated with attrition, to estimate participant-reported outcomes’ changes from baseline to follow-up (12 and 24 week means combined) [42]. Change estimates were transformed to effect sizes (Cohen’s *d*) using baseline estimates of pooled standard deviations. Change was estimated overall and by site. All analyses were conducted using SAS v9.4.

Due to the exploratory nature of the study, we relied on effect size estimation using Cohen’s guidelines for magnitude of effect size *d* (i.e. small: 0.2, moderate: 0.5, and large: 0.8) rather than hypothesis testing to interpret results; however we also report *p*-values for completeness.

## Results

### Sample characteristics

**Fig. 2:**
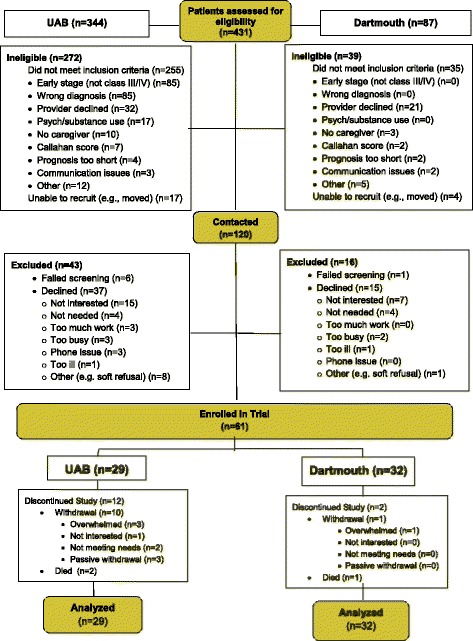
CONSORT diagram: Patient Recruitment, Treatment, and Analysis

We assessed 431 patients for eligibility (Fig. 2); approached 120 eligible patients for participation; and enrolled 61 patients (50% response rate) and 48 family caregivers. Eligible patients declined participation due to “not interested” (*n* = 22) or “not needed” (*n* = 8).

Overall, patients (*n* = 61) were a mean age of 71 years, male (51%; *n* = 31), white (80%, *n* = 49), Protestant (65.6%, *n* = 40), married or living with a partner (62%, *n* = 38), retired (56%, *n* = 34), were high school or General Education Diploma (GED) graduates (43%, *n* = 26) had Medicare/private insurance (64%, *n* = 39) and were rural (72.1%, *n* = 44) (Table 1). Patients and caregivers lived a median of 46 miles (range 1–177 miles) from UAB and 54 miles (range 6–128 miles) from DHMC. Compared to DHMC, UAB patients had higher proportions of black, Protestant, never married, and patients on disability. Clinically, most patients were NYHA Class IIIa/b or IV, with a mean ejection fraction of 38; 46% had no implanted cardiac device; 80% were on beta-blockers; and 59% were on statins. SHFM survival probabilities averaged 84% for 1-year, 72% for 2-years, and 49% for 5-years.

**Table 1:** Patient Demographics

	All patients (*N* = 61)	Dartmouth (*n* = 32)	UAB (*n* = 29)	*p* ^*^	*d* ^†^
	*n* (%)	*n* (%)	*n* (%)		
Age, Mean (SD)	70.59 (10.7)	73.41 (10.8)	67.48 (9.8)	0.03	0.55
Male	31 (50.8)	18 (56.2)	13 (44.8)	0.37	0.23
Race					
White	49 (80.3)	31 (96.9)	18 (62.1)		
Black	11 (18.0)	0 (0)	11 (37.9)	<.0001	0.95
Other	1 (1.6)	1 (3.1)	11 (37.9)		
Religion					
Protestant	40 (65.6)	13 (40.6)	27 (93.1)		
Catholic	10 (16.4)	8 (25.0)	2 (6.9)	<.0001	1.04
Other	4 (6.6)	4 (12.5)	0 (0)		
None	7 (11.5)	7 (21.9)	0 (0)		
Marital Status					
Never married	6 (9.8)	0 (0)	6 (20.7)		
Married or living with partner	38 (62.3)	21 (65.6)	17 (58.6)	0.04	0.55
Divorced or separated	4 (6.6)	3 (9.4)	1 (3.5)		
Widowed	13 (21.3)	8 (25.0)	5 (17.2)		
Work status					
Employed	6 (9.8)	3 (9.4)	3 (10.3)		
Retired/Homemaker	34 (55.7)	23 (71.9)	11 (37.9)	0.02	0.60
Not employed	3 (4.9)	0 (0)	3 (10.3)		
Disability	18 (29.5)	6 (18.8)	12 (41.4)		
Education					
< High school graduate	4 (6.6)	1 (3.1)	3 (10.3)		
High school graduate or GED	26 (42.6)	12 (37.5)	14 (48.3)		
Some college or technical school	15 (24.6)	9 (28.1)	6 (20.7)	0.42	0.21
College graduate	9 (14.8)	4 (12.5)	5 (17.2)		
Graduate degree	5 (8.2)	4 (12.5)	1 (3.5)		
No response	2 (3.3)	2 (6.3)	0 (0)		
Medical insurance					
Private/commercial	8 (13.1)	4 (12.5)	4 (13.8)		
Medicare/Medicaid	14 (23.0)	7 (21.9)	7 (24.1)	0.95	0.01
Medicare and Private	39 (63.9)	21 (65.6)	18 (62.0)		
NYHA class					
Class I	1 (1.6)	0 (0)	1 (3.5)	0.01	0.74
Class II	3 (4.9)	2 (6.3)	1 (3.5)		
Class IIIa	25 (41.0)	7 (21.9)	18 (62.0)		
Class IIIb	18 (29.5)	14 (43.8)	4 (13.8)		
Class IV	12 (19.7)	9 (28.1)	3 (10.3)		
Missing/Not recorded	2 (3.3)	0 (0)	2 (6.9)		
Ejection Fraction, Mean (SD)	37.86 (16.3)	36.81 (16.9)	39.05 (15.8)	0.60	0.14
Implanted Cardiac Devices					
None	28 (45.9)	10 (31.3)	18 (62.0)	0.16	0.36
BiV Pacemaker	7 (11.5)	5 (15.6)	2 (6.9)		
ICD	13 (21.3)	9 (28.1)	4 (13.8)		
BiV ICD	11 (18.0)	7 (21.9)	4 (13.8)		
Missing/Not recorded	2 (3.3)	1 (3.1)	1 (3.5)		
Heart Medications					
ACE-I	20 (32.8)	11 (34.4)	9 (31.0)	0.78	0.07
Beta-blocker	49 (80.3)	29 (90.6)	20 (69.0)	0.05	0.51
ARB	18 (29.5)	13 (40.6)	5 (17.2)	0.05	0.50
Statin	36 (59.0)	17 (53.1)	19 (65.5)	0.44	0.20
Allopurinol	8 (13.1)	5 (15.6)	3 (10.3)	0.71	0.10
Aldosterone blocker	27 (44.3)	17 (53.1)	10 (34.5)	0.20	0.33
None	3 (4.9)	1 (3.1)	2 (6.9)	0.60	0.14
Diuretic Intake (mg)					
Furosemide	29.02 (42.9)	32.19 (47.1)	25.52 (38.1)	0.55	0.16
Bumetanide	0.03 (0.3)	0 (0)	0.07 (0.4)	-	-
Torsemide	25.57 (44.0)	36.88 (48.9)	24.14 (38.6)	0.81	0.06
Metolazone	0.38 (1.2)	0 (0)	0.72 (1.7)	-	-
Hydrochlorothiazide	1.02 (4.7)	0.78 (4.4)	1.28 (5.1)	0.69	0.11
Lab data, *median*					
Hemoglobin (g/dL)	12.3 [9–16.4]	12.45 [9–16.4]	12.3 [10.1–16.2]	0.70	0.10
Lymphocyte %WBC	16.95 [3–48]	22.4 [3–48]	10 [4–42]	0.02	0.58
Uric acid (mg/dL)	6.65 [0–11]	7.25 [0–11]	2.05 [0–7.3]	0.01	1.15
Total cholesterol (mg/dL)	146 [0–253]	139 [0–253]	151 [0–259]	0.26	0.34
Sodium (mmol/L)	139 [123–145]	139.5 [123–145]	139 [127–145]	0.78	0.07
Seattle Heart Failure Model					
1-year survival probability, SD	0.84 (0.1)	0.82 (0.2)	0.87 (0.1)	0.13	0.38
2-year survival probability, SD	0.72 (0.2)	0.69 (0.2)	0.76 (0.1)	0.14	0.39
5-year survival probability, SD	0.49 (0.2)	0.45 (0.2)	0.53 (0.2)	0.18	0.36
Charlson Comorbidity Index	6.66 (92.5)	6.84 (1.8)	6.45 (3.0)	0.55	0.16
Rural Location	44 (72.1)	31 (96.9)	13 (44.8)	-	-

*Abbreviations*: *SD* standard deviation, *GED* General Education Development, *NYHA* New York Heart Association, *BiV* biventricular, *ICD* implantable cardioverter-defibrillator, *ACE-I* angiotensin-converting-enzyme inhibitor; ARB = angiotensin receptor blocker

^*^ *p*-values from t-test, Chi-squared, or Fisher’s exact tests, as appropriate

^†^Effect size: Cohen’s *d* or *d*-equivalent: small: *d* ~ 0.2, medium *d* ~ 0.5, large *d* ~ 0.8

Family caregivers (*n* = 48) were a mean age of 65 years, female (81%, *n* = 39), white (83%, *n* = 40), Protestant (52%, *n* = 25), married or living with a partner (81%, *n* = 39), retired (50%, *n* = 24), had a graduate degree (27%; *n* = 13) and were the patients’ spouse or partner (65%, *n* = 31) (Table 2). Compared to DHMC, UAB caregivers were more often black and Protestant.

**Table 2:** Caregiver Demographics

		All caregivers (*N* = 48)	Dartmouth (*n* = 29)	UAB (*n* = 19)		
		*n* (%)	*n* (%)	*n* (%)	*p* ^*^	*d* ^†^
Age, Mean (SD)		64.94 (9.3)	65.18 (10.3)	64.58 (7.7)	0.082	0.06
Gender						
Female		39 (81.3)	23 (79.3)	16 (84.2)	0.99	0
Race						
White		40 (83.3)	29 (100.0)	11 (57.9)	0.0002	1.19
Black		8 (16.7)	0 (0)	8 (42.1)		
Religion						
Protestant		25 (52.1)	7 (24.1)	18 (94.7)		
Catholic		7 (14.6)	7 (24.1)	0 (0)		
Jewish		1 (2.1)	1 (3.5)	0 (0)	<.0001	1.26
Other		6 (12.5)	6 (20.7)	0 (0)		
None		9 (18.8)	8 (27.6)	1 (5.3)		
Marital Status						
Never married		2 (4.2)	0 (0)	2 (10.5)	0.09	0.51
Married or living with partner		39 (81.3)	26 (89.7)	13 (68.4)		
Divorced or separated		3 (6.3)	2 (6.9)	1 (5.3)		
Widowed		4 (8.3)	1 (3.5)	3 (15.8)		
Work status						
Employed		17 (35.4)	9 (31.0)	8 (42.1)		
Retired/Homemaker		24 (50.0)	15 (51.7)	9 (47.4)		
Not employed		3 (6.3)	1 (3.5)	2 (10.5)	0.49	0.20
Disability		3 (6.3)	3 (10.3)	0 (0)		
No response		1 (2.1)	1 (3.5)	0 (0)		
Education						
<High school graduate		3 (6.3)	2 (6.9)	1 (5.3)		
High school graduate or GED		12 (25.0)	7 (24.1)	5 (26.3)		
Some college or technical school		11 (22.9)	5 (17.2)	6 (31.6)	0.23	0.36
College graduate		9 (18.8)	4 (13.8)	5 (26.3)		
Graduate degree		13 (27.1)	11 (37.9)	2 (10.5)		
Relationship to patient						
Spouse/partner		31 (64.6)	20 (69.0)	11 (57.9)		
Parent		8 (16.7)	4 (13.8)	4 (21.1)		
Son or daughter		4 (8..3)	2 (6.9)	2 (10.5)	0.63	0.14
Other relative		3 (6.3)	1 (3.5)	2 (10.5)		
Friend		2 (4.2)	2 (6.9)	0 (0)		

*Abbreviations*: *SD* standard deviation, *GED* General Education Development

^*^ *p*-values from t-test or Fisher’s exact tests, as appropriate

^†^Effect size: Cohen’s *d* or *d*-equivalent; small: *d* ~ 0.2, medium *d* ~ 0.5, large *d* ~ 0.8

### Feasibility/acceptability: Intervention and measure completion

Overall study attrition was due to withdrawal (18%; *n* = 11) and death (5%; *n* = 3). In-person comprehensive palliative care assessments were completed by 64% (*n* = 39) (UAB = 41%; *n* = 12 vs. DHMC 84.4%; *n* = 27). Non-completion (*n* = 22) was due to “declined” (61%; *n* = 14), “no-show” (26%; *n* = 6) or died before appointment (13%; *n* = 2). Most patients (69%; *n* = 42; UAB = 41%; *n* = 12 vs DHMC = 94%; *n* = 30) and caregivers (79%; *n* = 38; UAB = 63%; *n* = 12 vs DHMC = 90%; *n* = 26) completed the nurse coaching sessions. Average weekly session duration was 46- (caregivers)–50-(patients) minutes and monthly check-in calls were 13 min. At the completion of the weekly sessions, nurse coaches assessed patient and caregiver satisfaction with the intervention, which was high and no participants reported adverse events.

We observed between-site differences in measurement completion for patients (UAB = 38%; *n* = 11 vs DHMC =72%; *n* = 23; *p* = 0.008, *d*-equivalent = 0.7), but not for caregivers (UAB = 58%; *n* = 11 vs DHMC = 69%; *n* = 20; *p* = 0.54, *d*-equivalent = 0.18).

Exploratory analyses of study attrition, and participant baseline demographics and outcomes (Additional file 1: Tables S2 and S3) revealed that the strongest predictors of patient attrition were site (UAB vs. DHMC: OR = 4.7, 95% C.I. = [1.6, 14.4], *p* = 0.006) and baseline Patient Assessment of Chronic Illness Care (PACIC)-patient activation subscale score (OR = 0.57 per SD increase, 95% C.I. = [0.3, 0.9], *p* = 0.026). The strongest predictor of caregiver attrition was decreased caregiver QOL (measured by the Bakas Caregiving Outcomes Scale (BCOS) score) (OR = 0.49 per SD increase, 95% C.I. = [0.2, 1.1], *p* = 0.073).

Patient and caregiver feedback relative to the intervention included the density, high literacy level of the patient/caregiver Charting Your Course guides, and difficulty attending (due to travel distance/transportation) and misunderstanding the purpose of the in-person outpatient palliative care consultation. They also provided critical feedback about the study measures: they reported an inability to complete the literacy measure, and a high burden of completing the symptom measure.

### Patient reported outcome measures

Key effect size differences were evident between the UAB and DHMC patient-reported baseline (Additional file 1: Table S4) and change from baseline to week 24 outcomes (Table 3). At baseline, relative to DHMC, UAB had >0.20 effect size differences that were lower in KCCQ symptom subscale, PROMIS mental health, PACIC (activation scale) and humor coping and higher KCCQ-QOL subscale, perceived social support (e.g. MSPSS) and use of denial and religious coping strategies. UAB patients experienced moderate improvements post-intervention (baseline to 24-week) in all KCCQ subscales (d = 0.37–0.79), MSAS-HF symptom burden index, HADS (d = 0.31–0.34), and Physical and Mental Global Health subscales (d = 0.46–0.53) and a small magnitude of improvement in the decision-support subscale. DHMC patients had a moderate post-intervention improvement in the MSAS-HF symptom burden index (d = .50) and small-moderate improvements (but to a lesser extent than UAB) in the KCCQ (d = 0.21–0.39), HADS (d = 0.20–0.23) and Global Mental Health (d = 0.23) subscales.

**Table 3:** Patient-reported Outcome Measures - Change from Baseline (Adjusted)

	All patients			Dartmouth			UAB		
	Mean (SE)	*p* ^*^	Effect size ^†^	Mean (SE)	*p* ^*^	Effect size ^†^	Mean (SE)	*p* ^*^	Effect size ^†^
KCCQ									
Physical limitation	13.30 (4.4)	0.003	0.50	9.90 (6.1)	0.11	0.37	17.90 (6.2)	0.01	0.67
Symptoms	10.80 (4.3)	0.01	0.44	5.30 (5.5)	0.34	0.21	17.00 (6.6)	0.01	0.69
Social limitation	8.40 (5.1)	0.10	0.28	6.20 (6.7)	0.36	0.21	11.10 (7.8)	0.16	0.37
Quality of life	10.70 (4.0)	0.009	0.4	10.40 (5.4)	0.06	0.39	11.30 (6.2)	0.07	0.42
KCCQ functional status	11.60 (4.0)	0.005	0.49	6.90 (5.3)	0.20	0.29	17.50 (5.8)	0.004	0.74
KCCQ clinical summary	10.30 (3.9)	0.009	0.44	7.80 (5.2)	0.14	0.33	13.70 (5.7)	0.02	0.58
MSAS-HF Symptom Burden Index	−25.80 (7.1)	0.0004	−0.54	−24.20 (8.0)	0.003	−0.50	−25.7 (12.8)	0.05	−0.54
HADS									
Anxiety	−1.00 (0.5)	0.08	−0.29	−0.70 (0.6)	0.28	−0.20	−1.20 (0.9)	0.20	−0.34
Depression	−1.10 (0.6)	0.07	−0.28	−0.90 (0.7)	0.17	−0.23	−1.20 (1.1)	0.28	−0.31
PROMIS									
Global Physical Health T score	2.70 (1.5)	0.08	0.32	1.50 (2.0)	0.47	0.18	3.80 (2.0)	0.07	0.46
Global Mental Health T score	3.00 (1.4)	0.04	0.36	1.90 (1.8)	0.30	0.23	4.40 (2.2)	0.05	0.53
PACIC									
Patient activation	0.20 (0.2)	0.26	0.17	0.20 (0.2)	0.35	0.17	0 (0.3)	0.95	0
Decision support	0.30 (0.2)	0.10	0.30	0.20 (0.2)	0.45	0.2	0.30 (0.3)	0.23	0.30
Goal setting	0.30 (0.2)	0.09	0.29	0.30 (0.2)	0.25	0.29	0.20 (0.2)	0.48	0.20
Problem solving	0.30 (0.2)	0.14	0.27	0.20 (0.2)	0.29	0.18	0.20 (0.3)	0.59	0.18
Care Coordination	0 (0.2)	0.92	0	−0.10 (0.2)	0.61	−0.09	0.10 (0.3)	0.80	0.09
PACIC Summary Score	0.20 (0.1)	0.14	0.23	0.10 (0.2)	0.42	0.11	0.10 (0.2)	0.50	0.11

*Abbreviations*: *SE* standard error, *KCCQ* Kansas City Cardiomyopathy Questionnaire, *MSAS-HF* Memorial Symptom Assessment Scale-Heart Failure, *HADS* Hospital Anxiety and Depression Scale, *PROMIS* Patient Reported Outcomes Measurement Information System, *PACIC* Patient Assessment of Chronic Illness Care. All change from baseline estimates were adjusted for measures associated with attrition (e.g. religious preference, baseline PACIC-Patient Activation subscale and SHFM 1-year survival probability)

^*^ *p*-values from t-test or Fisher’s exact tests, as appropriate

^†^Effect size: Cohen’s *d* or *d*-equivalent; small: *d* ~ 0.2, medium *d* ~ 0.5, large *d* ~ 0.8

### Caregiver reported outcome measures

No between-site differences were noted in caregiver-reported baseline outcomes (Additional file 1: Table S5). However post-intervention estimates of change from baseline to 24 weeks demonstrated that UAB caregivers had moderate effect size improvements in BCOS, HADS-Depression, Global Mental Health, and MBCB scores and small magnitude improvements in HADS-Anxiety and Global Physical Health (Table 4). At DHMC the moderate post-intervention improvement was noted for MBCB-Stress Burden and MBCB total scores and small improvements in BCOS, HADS–depression and PAC-outlook on life subscales.

**Table 4:** Caregiver-reported Outcomes - Change from Baseline to 24 weeks (Adjusted for BCOS)

	All caregivers			Dartmouth			UAB		
	Mean (SE)	*p* ^*^	Effect size^†^	Mean (SE)	*p* ^*^	Effect size^†^	Mean (SE)	*p* ^*^	Effect size^†^
BCOS score	3.70 (2.0)	0.07	0.40	2.30 (2.2)	0.30	0.25	6.70 (3.8)	0.09	0.73
HADS									
Anxiety	−0.20 (0.5)	0.69	−0.07	0.10 (0.7)	0.86	0.03	−0.70 (0.9)	0.42	−0.23
Depression	−1.30 (0.7)	0.08	−0.32	−1.10 (0.9)	0.26	−0.27	−1.90 (1.1)	0.11	−0.47
PROMIS									
Global Physical Health	1.70 (1.4)	0.21	0.22	1.60 (1.8)	0.38	0.2	1.60 (2.2)	0.45	0.20
Global Mental Health	1.80 (1.3)	0.18	0.24	1.00 (1.8)	0.57	0.13	3.20 (1.8)	0.08	0.43
MBCB									
Objective burden	−1.10 (0.5)	0.02	−0.33	−0.50 (0.6)	0.39	−0.15	−2.10 (0.8)	0.01	−0.63
Demand burden	−0.60 (0.4)	0.09	−0.28	−0.04 (0.3)	0.22	−0.19	−1.20 (0.8)	0.16	−0.56
Stress burden	−1.30 (0.4)	0.001	−0.58	−1.40 (0.5)	0.003	−0.62	−1.40 (0.7)	0.07	−0.62
Total Score	−3.10 (1.0)	0.002	−0.53	−2.40 (1.0)	0.03	−0.41	−4.60 (2.0)	0.03	−0.78
PAC									
Self-affirmation	0.40 (0.8)	0.58	0.11	0.30 (0.9)	0.70	0.08	0.60 (1.4)	0.68	0.16
Outlook on life	0.40 (0.4)	0.41	0.16	0.50 (0.6)	0.41	0.20	0.40 (0.7)	0.61	0.16
PAC Total	0.90 (1.2)	0.43	0.15	0.90 (1.4)	0.52	0.15	1.10 (2.0)	0.57	0.19

*Abbreviations*: *SE* standard error, *BCOS* Bakas Caregiving Outcomes Scale, *HADS* Hospital Anxiety and Depression Scale, *PROMIS* Patient Reported Outcomes Measurement Information System, *MBCB* Montgomery Borgatta Caregiver Burden Scale, *PAC* Positive Aspects of Caregiving

^*^ *p*-values from t-test or Fisher’s exact tests, as appropriate

^†^Effect size: Cohen’s *d* or *d*-equivalent; small: *d* ~ 0.2, medium *d* ~ 0.5, large *d* ~ 0.8

All change estimates were adjusted for the baseline measures most strongly associated with caregiver attrition: caregiver education, baseline BCOS score and baseline MBCB - objective burden subscale

### Resource use

At baseline there were no between-group differences in hospital days, intensive care unit (ICU) days or emergency department (ED) visits (Table 5). However, from baseline to 24 weeks, a small-moderate effect size decrease was noted in hospital and ICU days per month; a small effect size decrease in ED visits per month was only noted at UAB. At baseline 87% (*n* = 28) of DHMC and 28% (*n* = 8) of UAB patients had an advance directive (*p* < 0.001); by study end, one additional patient per site completed an advance directive. Related to hospice care, at baseline each site had one patient enrolled in hospice and by study end, four additional patients were enrolled in hospice (UAB = 1; DHMC = 3) (*p* = 0.07; d = 0.48).

**Table 5:** Resource Use At Baseline

At Baseline (prior 3 months)	All patients (*N* = 60)			Dartmouth (*n* = 31)			UAB (*n* = 29)		
	Mean (SD)			Mean (SD)			Mean (SD)	*p*	Effect size
Hospital days/month	1.44 (3.0)			1.7 (3.6)			1.17 (2.2)	0.72	0.18
ICU days/month	0.26 (1.0)			0.34 (1.3)			0.16 (0.6)	0.32	0.18
ED visits/month	0.23 (0.4)			0.26 (0.4)			0.21 (0.4)	0.54	0.13
Resource Use- Difference from Baseline									
	All patients (*N* = 34)			Dartmouth (*n* = 23)			UAB (*n* = 11)		
	Mean (SE)	*p* ^*^	Effect size^†^	Mean (SE)	*p* ^*^	Effect size^†^	Mean (SE)	*p* ^*^	Effect size^†^
Hospital days/month	−0.89 (0.3)	0.002	0.39	−1.18 (0.4)	0.006	0.57	−0.61 (0.4)	0.13	0.24
Days/month in ICU, Mean	−0.16 (0.1)	0.06	0.29	−0.12 (0.1)	0.23	0.26	−0.21 (0.2)	0.16	0.31
ED visits/month, Mean	−0.05 (0.1)	0.33	0.17	0 (0.1)	0.94	0.05	−0.09 (0.1)	0.26	0.26

*Abbreviations*: *SD* standard deviation, *SE* standard error, Estimates from longitudinal models fitted with negative binomial distributions (log link), adjusted for baseline, *PACIC* Patient Activation, and religious preference

^*^ *p*-values from t-test or Fisher’s exact tests, as appropriate

^†^Effect size: Cohen’s d (Cohen, 1988), or d-equivalent (Rosenthal & Rubin, 2003) small: d ~ 0.2, moderate d ~ 0.5, large d ~ 0.8

## Discussion

The purpose of this 2-site, single-arm pilot study was to determine feasibility and potential efficacy of implementing the ENABLE CHF-PC EPC tele-health intervention in a racially-diverse, southeastern US HF population. Previously, ENABLE had demonstrated effectiveness in two large cancer RCTs [33, 43] and in a small, mostly white northeastern HF sample [30]. We were able to achieve our primary study feasibility/acceptability aim in a racially and culturally diverse sample by engaging patients and family caregivers and soliciting their feedback to make improvements in the study design, measures, and intervention.

The key lessons learned from this pilot could be of considerable value to other researchers and clinicians attempting to integrate supportive and palliative care into racially-diverse HF populations. First, health literacy issues were marked in our trial and resulted in changes to future study outcome measures, intervention materials, and recruitment and retention procedures. Per our study coordinator reports, participants expressed frustration and dissatisfaction in completing our original health literacy measure, The Newest Vital Sign [44]. We recommend that others be sensitive to health literacy when working with this population and consider pretesting all measures and materials prior to initiating them in large scale trials.

Second, we encountered significant recruitment challenges in the southeastern site. We needed to screen more UAB individuals for eligibility (*n* = 344) compared to DHMC (*n* = 87) and proportionally fewer eligible UAB patients agreed to participate. Several factors may explain this discrepancy. The southeast has a high proportion of individuals of black race; this population experiences the highest burden of illness from HF in the US and at a much younger age than whites [45]. Blacks have also been noted to have high rates of healthcare system mistrust and a much lower uptake of palliative and hospice services than whites [46–51]. In response to these factors, we reduced our age eligibility criterion from 65 to 50 years and contracted with a recruitment service who was highly-experienced in community-based research in racially underserved populations and maintained weekly communications with the recruitment service and UAB cardiologists at HF clinical meetings.

Second, this was our first effort to identify and recruit eligible, racially-diverse HF patients for an early palliative care study. Our cardiologist co-investigators were extremely supportive and cooperative, and assisted us to find the most efficient and effective way to identify eligible patients and refine our screening, recruitment and operational procedures without disrupting clinical patient care. Early on, we also realized the need to adjust processes to improve UAB participants’ uptake of the outpatient palliative care consultation component of the intervention. The UAB Supportive Care and Survivorship Clinic faculty and staff helped us to refine outpatient palliative care consultation scheduling procedures so that, when-ever possible, these visits would coincide with other ap-pointments in an effort to overcome transportation issues of patients who lived long distances from the medical center. Many UAB patients lacked familiarity or had misperceptions about EPC, in some cases confusing it with hospice care. We provided our recruiters with ex-tensive training about the goals of EPC as providing “an extra layer of support” so that they were better able to introduce the study in a non-threatening way. We lever-aged the trusting relationship that most HF patients had with their cardiologists and the UAB health system and carefully ‘branded’ our materials to be consistent with all other UAB programs. We have also expanded our in-progress RCT recruitment sites to include the local Veteran’s Affairs Medical Center, a nurse-led clinic for under-insured HF patients, and we are developing partnerships with community agencies, payers, and churches. Fostering clinical [52] and community [48, 52–54] relationships are critical components of successful community-based recruitment.

Third, participant retention was equally challenging. As is common in palliative care trials, [55, 56] both sites experienced considerable intervention, measurement and overall attrition. Fewer UAB patients participated in the outpatient palliative care consultation (32% vs 68%) and intervention sessions (41% vs 94%), and fewer UAB caregivers completed all intervention sessions (63% vs 90%). In exploratory analyses, in addition to site, we found links between patient attrition and lower activation scores and between caregiver attrition and lower QOL scores. We offer two explanations for our differential attrition. First, during joint weekly supervisory meetings, UAB more so than DHMC nurse coaches, reported that participants found the *Charting Your Course* guides to be lengthy and text-heavy. This combined with lower ‘activation’ levels may have caused some UAB participants to find the intervention burdensome. We revised our materials to be more colorful and pictorial to address what may have been a health literacy issue. The link between caregiver attrition and quality of life and burden is not surprising given that caregivers have been shown to neglect their own needs in favor of caring for the patient. Hence caregivers may not make time for participation in a support intervention [57]. Of interest, distance to the centers and patients’ disability status were not predictors of attrition, reinforcing the ability of telephonic services to increase palliative care access.

Though not powered for hypothesis testing, we identified small-to-moderate longitudinal improvements in QOL, symptom, and psychological patient and caregiver-reported outcomes. Of interest is that there was a more robust improvement noted in UAB vs. DHMC patients and caregivers. The higher attrition at UAB may account for this difference. We were also intrigued by the significant between-site differences in the patient activation measure; UAB patients had much lower activation scores than DHMC patients and the scores remained stable over the course of the study. In our prior cancer study we also did not see a signal in this measure [33]. Though others have found improvement in PACIC empowerment subscales from early initiation of supportive care (via nurse navigators) [58] it may be that ‘activation’ is not the EPC mechanism or that the PACIC is not sufficiently sensitive to detect changes in activation. Alternatively, the relatively high activation scores observed in the DHMC cancer and HF samples, may be an indication that the instrument has a ceiling effect.

Several limitations of these findings are important to note. First, as a single-arm feasibility pilot study, the trial did not have a control group and was not powered to evaluate efficacy. However, as our primary goal was feasibility in a culturally-diverse clinical setting and population, we learned valuable lessons that informed our subsequent RCT, thereby reinforcing the necessary step of pilot testing interventions prior to embarking on large-scale intervention trials [59]. Second, the patient and caregiver identified post-intervention improvements are inconclusive, preliminary, and not to be generalized as eligibility criteria and intervention changes were made during the course of the pilot. Differential attrition between the two sites may account entirely for the outcome differences. We are currently conducting a large, NIH/NINR-funded clinical trial of ENABLE CHF-PC to evaluate efficacy and address most of these limitations.

## Conclusion

In conclusion, a model of concurrent HF palliative care was feasibly pilot-tested in a heterogeneous sample of individuals with NYHA Class III/IV HF and their family caregivers. Between-site demographic, attrition, and participant-reported outcomes highlight the importance of intervention pilot-testing in culturally-diverse populations.

## Additional file

Additional file 1: Table S1. Description of Primary Outcome Measures. **Table S2.** Association Between Patient Baseline Characteristic and Attrition (*N* = 61). **Table S3.** Association Between Caregiver Baseline Characteristic and Attrition (*N* = 48). **Table S4.** Patient-Reported Outcomes- Baseline. **Table S5.** Caregiver-Reported Outcomes- Baseline. (DOCX 30 kb)
