# Supplementary material for: Engaging patients and families to create a feasible clinical trial integrating palliative and heart failure care: results of the ENABLE CHF-PC pilot clinical trial
Source: BMC Palliat Care. 2017 Aug 31;16:45. doi: 10.1186/s12904-017-0226-8 (PMC5580310; doi:10.1186/s12904-017-0226-8)
Supplement: Additional file 1: Table S1. — Description of Primary Outcome Measures. Table S2. Association Between Patient Baseline Characteristic and Attrition (N = 61). Table S3. Association Between Caregiver Baseline Characteristic and Attrition (N = 48). Table S4. Patient-Reported Outcomes- Baseline. Table S5. Caregiver-Reported Outcomes- Baseline. (DOCX 30 kb) [file 12904_2017_226_MOESM1_ESM.docx]

| **Supplemental Table 1.** Description of Primary Outcome Measures | | |
| --- | --- | --- |
| **Instrument** | **Description** | **Reliability** |
| **Patients with Heart Failure** | | |
| Kansas City Cardiomyopathy Questionnaire (KCCQ) | 23 items; quality of life measure with 5 domains: physical limitations, symptoms, self-efficacy, social interference, and QOL | Subscale α=0.62-0.90 |
| Memorial Symptom Assessment Scale-Heart Failure (MSAS-HF) Symptom Burden | 32 items; symptom frequency, severity and distress. Symptom burden is the overall sum of the number of symptoms present | Subscales α=0.73-0.92 |
| Hospital Anxiety and Depression Scale (HADS) | 14 items; 2 domains measuring depression and anxiety; higher scores=higher depression and anxiety | Subscales α=0.82-0.93 |
| Patient Reported Outcomes Measurement Information System (PROMIS) Short Form v.1.0-1.1 Global(60,61) | 10 items; overall health measure; items address physical health, mental health, ability to carry out usual social and physical activities, fatigue, pain; higher scores=higher overall health | Subscales α=0.81-0.86 |
| Patient Assessment of Chronic Illness Care (PACIC) | 20 items; 5 constructs: patient activation, delivery system/ decision support, goal setting, problems solving, and follow-up coordination | Subscales α=0.78-0.90 |
| Multidimensional Scale of Perceived Social Support (MSPSS) | 12 items; 3 subscales measures perceived adequacy of social support from significant other, family, and friends; higher overall summary score=higher levels of perceived support | High internal test-retest reliability |
| Brief COPE | 28 items measuring 14 different coping strategies: self-distraction, active coping, substance abuse, emotional support, instrumental support, planning, positive reframing, denial, behavioral disengagement, venting, humor, acceptance, religion, self-blame. | Subscales α=0.79-0.68 |
| **Family Caregivers** | | |
| Bakas Caregiving Outcomes Scale (BCOS)(62) | 15 items; quality of life measure; items address self-esteem, ability to cope, relationship with friends, emotional wellbeing, future outlook, general health, financial wellbeing; higher scores=better QOL | α=0.90 |
| Hospital Anxiety and Depression Scale (HADS) | *Same as above* | *Same as above* |
| PROMIS Short Form v.1.0-1.1 Global | *Same as above* | *Same as above* |
| Montgomery Borgatta Caregiver Burden Scale (MBCB)(63) | 35 items; burden measure with 3 domains: objective burden, stress burden, and demand burden; higher scores=higher burden | Subscales α=0.75-0.88 |
| Positive Aspects of Caregiving (PAC) | 9 items; measures caregiver’s perceived benefits of caregiving with 2 subscales: self-affirmation and outlook. | Subscales α =0.86-0.80; overall=0.89 |
| Note: Patients and caregivers also were asked to complete the Newest Vital Sign Literacy Measure however only 30% were able, so that data is not included. | | |

| **Supplemental Table 2.** Association Between Patient Baseline Characteristic and Attrition (N=61) | | | | |
| --- | --- | --- | --- | --- |
| Instrument | Measure or subscale | Odds Ratio of attrition per SD increase | | |
|  |  | Estimate | 95% | p |
| Demographics | Age | 0.73 | 0.43, 1.23 | 0.24 |
| Demographics | Distance from site | 1.17 | 0.70, 1.96 | 0.54 |
| Chart review | SHFM 1-year survival probability | 1.69 | 0.82, 3.49 | 0.16 |
| KCCQ | Physical limitation | 0.83 | 0.49, 1.41 | 0.50 |
| KCCQ | Symptoms | 0.80 | 0.47, 1.35 | 0.40 |
| KCCQ | Social limitation | 1.01 | 0.60, 1.69 | 0.98 |
| KCCQ | Quality of life | 1.24 | 0.73, 2.09 | 0.43 |
| KCCQ | KCCQ functional status | 0.84 | 0.50, 1.42 | 0.51 |
| KCCQ | KCCQ clinical summary | 1.02 | 0.61, 1.72 | 0.93 |
| MSAS | Symptom burden index | 1.12 | 0.67, 1.87 | 0.67 |
| HADS | Anxiety | 0.98 | 0.58, 1.64 | 0.93 |
| HADS | Depression | 1.00 | 0.59, 1.67 | 0.99 |
| PROMIS | Global Physical Health T score | 0.97 | 0.58, 1.63 | 0.91 |
| PROMIS | Global Mental  Health T score | 1.30 | 0.77, 2.19 | 0.33 |
| PACIC | Patient activation | 0.53 | 0.30, 0.93 | 0.03 |
| PACIC | Decision support | 0.67 | 0.39, 1.14 | 0.14 |
| PACIC | Goal setting | 0.83 | 0.49, 1.40 | 0.49 |
| PACIC | Problem solving | 0.71 | 0.42, 1.21 | 0.21 |
| PACIC | Care Coordination | 0.85 | 0.51, 1.44 | 0.55 |
| MSPSS | Significant other | 0.76 | 0.46, 1.28 | 0.31 |
| MSPSS | Family | 0.81 | 0.49, 1.36 | 0.43 |
| MSPSS | Friends | 0.8 | 0.48, 1.35 | 0.40 |
| MSPSS | Total MSPSS score | 0.76 | 0.45, 1.28 | 0.31 |
| Brief COPE | Self-distraction | 0.86 | 0.51, 1.44 | 0.56 |
| Brief COPE | Active coping | 0.99 | 0.59, 1.66 | 0.97 |
| Brief COPE | Denial | 0.98 | 0.58, 1.64 | 0.93 |
| Brief COPE | Substance use | 0.92 | 0.53, 1.59 | 0.75 |
| Brief COPE | Use of emotional support | 0.87 | 0.52, 1.45 | 0.60 |
| Brief COPE | Use of instrumental support | 0.87 | 0.52, 1.46 | 0.60 |
| Brief COPE | Behavioral disengagement | 0.82 | 0.47, 1.45 | 0.50 |
| Brief COPE | Venting | 0.94 | 0.56, 1.59 | 0.82 |
| Brief COPE | Positive reframing | 1.08 | 0.64, 1.81 | 0.77 |
| Brief COPE | Planning | 1.31 | 0.76, 2.25 | 0.33 |
| Brief COPE | Humor | 0.88 | 0.52, 1.48 | 0.63 |
| Brief COPE | Acceptance | 1.49 | 0.86, 2.60 | 0.16 |
| Brief COPE | Religion | 1.57 | 0.91, 2.70 | 0.11 |
| Brief COPE | Self-blame | 1.03 | 0.61, 1.72 | 0.91 |
| SD = standard deviation; CL = confidence level; KCCQ = Kansas City Cardiomyopathy Questionnaire; MSAS-HF = Memorial Symptom Assessment Scale-Heart Failure; HADS = Hospital Anxiety and Depression Scale; PROMIS = Patient Reported Outcomes Measurement Information System; PACIC = Patient Assessment of Chronic Illness; PACIC = Patient Assessment of Chronic Illness; MSPSS = Multidimensional Scale of Perceived Social Support.  Odds ratios as standardized effect sizes (Chinn, 2000): small: OR ~1.2 or 0.83, for an SD increase; medium: OR ~1.72 or 0.58, for an SD increase; large: OR: 2.48 or 0.4, for an SD increase | | | | |

| **Supplemental Table 3.** Association Between Caregiver Baseline Characteristic and Attrition (N=48) | | | | |
| --- | --- | --- | --- | --- |
| Instrument | Baseline Measure or subscale | Odds Ratio of attrition per SD increase | | |
|  |  | Estimate | 95% CL | p |
| BCOS | BCOS score | 0.49 | 0.23, 1.07 | 0.07 |
| HADS | Anxiety | 1.15 | 0.64, 2.08 | 0.64 |
| HADS | Depression | 1.52 | 0.83, 2.78 | 0.18 |
| PROMIS | Global Physical Health T score | 0.69 | 0.37, 1.28 | 0.24 |
| PROMIS | Global Mental Health T score | 0.69 | 0.37, 1.29 | 0.24 |
| MBCB | Objective burden | 1.60 | 0.86, 2.97 | 0.14 |
| MBCB | Demand burden | 1.19 | 0.65, 2.18 | 0.57 |
| MBCB | Stress burden | 1.06 | 0.58, 1.92 | 0.86 |
| MBCB | MBCB Total | 1.43 | 0.76, 2.69 | 0.26 |
| PAC | Self-affirmation | 0.78 | 0.42, 1.42 | 0.41 |
| PAC | Outlook on life | 0.72 | 0.39, 1.32 | 0.29 |
| PAC | PAC Total | 0.72 | 0.39, 1.32 | 0.29 |
| SD = standard deviation; CL = confidence level; BCOS = Bakas Caregiving Outcomes Scale; HADS = Hospital Anxiety and Depression Scale; PROMIS = Patient Reported Outcomes Measurement Information System; MBCB = Montgomery Borgatta Caregiver Burden Scale; PAC =  Positive Aspects of Caregiving | | | | |
| Odds ratios as standardized effect sizes (Chinn, 2000): small: OR ~1.2 or 0.83, for an SD increase; medium: OR ~1.72 or 0.58, for an SD increase; large: OR: 2.48 or 0.4, for an SD increase. | | | | |

| **Supplemental Table 4.** Patient-Reported Outcomes- Baseline | | | | | | |
| --- | --- | --- | --- | --- | --- | --- |
|  |  | All patients (N=48) | Dartmouth (n=29) | UAB (n=19) |  |  |
|  |  | Mean (SD) | Mean (SD) | Mean (SD) | p^*^ | Effect size^†^ |
| KCCQ | |  |  |  |  |  |
| Physical limitation | | 49.08 (26.8) | 47.18 (27.8) | 51.12 (25.9) | 0.57 | 0.15 |
| Symptoms | | 53.23 (24.8) | 56.54 (24.2) | 49.58 (25.3) | 0.28 | 0.28 |
| Social limitation | | 58.71 (30.0) | 56.64 (29.5) | 60.99 (30.8) | 0.58 | 0.15 |
| Quality of life | | 60.38 (26.6) | 57.03 (27.7) | 64.08 (25.3) | 0.31 | 0.27 |
| KCCQ functional status | | 51.57 (23.7) | 52.68 (23.9) | 50.35 (23.9) | 0.70 | 0.10 |
| KCCQ clinical summary | | 56.89 (23.6) | 55.45 (24.3) | 58.47 (23.0) | 0.62 | 0.13 |
| MSAS-HF Symptom Burden Index | | 83.00 (48.0) | 83.78 (42.5) | 82.14 (54.1) | 0.90 | 0.03 |
| HADS | |  |  |  |  |  |
| Anxiety | | 5.72 (3.5) | 5.75 (3.1) | 5.69 (4.0) | 0.95 | 0.02 |
| Depression | | 4.28 (3.9) | 4.00 (3.1) | 4.59 (4.7) | 0.57 | 0.15 |
| PROMIS Global Health | |  |  |  |  |  |
| Physical | | 39.25 (8.3) | 39.91 (9.1) | 38.53 (7.6) | 0.52 | 0.17 |
| Mental | | 45.45 (8.3) | 46.41 (8.4) | 44.39 (8.4) | 0.35 | 0.24 |
| PACIC | |  |  |  |  |  |
| Patient activation | | 3.17 (1.2) | 3.66 (1.1) | 2.63 (1.0) | 0.0004 | 0.87 |
| Decision support | | 3.66 (0.99) | 3.93 (0.9) | 3.37 (1.1) | 0.03 | 0.57 |
| Goal setting | | 2.96 (1.02) | 3.29 (1.0) | 2.6 (0.9) | 0.01 | 0.68 |
| Problem solving | | 3.58 (1.1) | 3.94 (0.9) | 3.18 (1.2) | 0.01 | 0.68 |
| Care Coordination | | 2.43 (1.1) | 2.81 (1.0) | 2.02 (1.0) | 0.003 | 0.75 |
| PACIC Summary Score | | 3.09 (0.9) | 3.45 (0.8) | 2.69 (0.8) | 0.0005 | 0.87 |
| MSPSS ^‡^ | |  |  |  |  |  |
| Significant other | | 23.90 (4.1) | 23.50 (4.7) | 24.33 (3.5) | 0.43 | 0.20 |
| Family | | 23.30 (5.0) | 22.09 (6.1) | 24.62 (2.9) | 0.05 | 0.51 |
| Friends | | 21.82 (4.4) | 20.78 (4.6) | 22.97 (4.0) | 0.05 | 0.50 |
| Total MSPSS score | | 69.00 (11.5) | 66.38 (12.6) | 71.90 (9.7) | 0.06 | 0.48 |
| Brief Cope ^‡^ | |  |  |  |  |  |
| Self-distraction | | 1.54 (0.9) | 1.61 (0.7) | 1.47 (1.0) | 0.52 | 0.16 |
| Active coping | | 2.02 (0.9) | 2.00 (0.9) | 2.05 (1.0) | 0.83 | 0.05 |
| Denial | | 0.43 (0.8) | 0.34 (0.7) | 0.53 (0.9) | 0.35 | 0.24 |
| Substance use | | 0.01 (0.1) | 0.02 (0.1) | 0 (0) | - | - |
| Use of emotional support | | 2.16 (1.0) | 2.11 (1.0) | 2.22 (0.9) | 0.64 | 0.12 |
| Use of instrumental support | | 1.46 (1.0) | 1.47 (1.0) | 1.45 (1.0) | 0.94 | 0.02 |
| Behavioral disengagement | | 0.27 (0.6) | 0.23 (0.6) | 0.31 (0.6) | 0.63 | 0.13 |
| Venting | | 0.78 (0.7) | 0.77 (0.7) | 0.79 (0.7) | 0.88 | 0.03 |
| Positive reframing | | 1.48 (1.0) | 1.53 (1.1) | 1.41 (0.9) | 0.64 | 0.12 |
| Planning | | 2.04 (0.9) | 2.08 (0.9) | 2.00 (0.9) | 0.73 | 0.09 |
| Humor | | 0.84 (1.0) | 0.94 (1.0) | 0.72 (1.0) | 0.41 | 0.22 |
| Acceptance | | 2.44 (0.7) | 2.48 (0.7) | 2.40 (0.7) | 0.61 | 0.12 |
| Religion | | 1.78 (1.2) | 1.13 (1.1) | 2.50 (0.9) | <.0001 | 1.13 |
| Self-blame | | 0.74 (0.9) | 0.80 (0.9) | 0.67 (0.9) | 0.59 | 0.14 |
| SD = standard deviation; KCCQ = Kansas City Cardiomyopathy Questionnaire; MSAS-HF = Memorial Symptom Assessment Scale-Heart Failure; HADS = Hospital Anxiety and Depression Scale; PROMIS = Patient Reported Outcomes Measurement Information System; PACIC = Patient Assessment of Chronic Illness; MSPSS = Multidimensional Scale of Perceived Social Support.  ^*^ p-values from t-test or Fisher's exact tests, as appropriate.  ^†^ Effect size: Cohen's d (Cohen, 1988), or d-equivalent (Rosenthal & Rubin, 2003) small: d~0.2, medium d~0.5, large d~0.8.  ^‡^ Measure collected at baseline only. | | | | | | |

| **Supplemental Table 5.** Caregiver-Reported Outcomes- Baseline | | | | | | |
| --- | --- | --- | --- | --- | --- | --- |
|  |  | All caregivers (N=48) | Dartmouth (n=29) | UAB (n=19) |  |  |
|  |  | Mean (SD) | Mean (SD) | Mean (SD) | p ^*^ | Effect size^†^ |
| BCOS score | | 58.21 (9.2) | 57.17 (7.3) | 59.79 (11.6) | 0.39 | 0.28 |
| HADS | |  |  |  |  |  |
| Anxiety | | 3.6 (3.0) | 3.31 (2.8) | 4.05 (3.4) | 0.43 | 0.25 |
| Depression | | 5.69 (4.0) | 5.79 (4.0) | 5.53 (4.2) | 0.83 | 0.06 |
| PROMIS Global Health | |  |  |  |  |  |
| Physical | | 19.56 (3.1) | 19.7 (3.4) | 19.34 (2.8) | 0.70 | 0.12 |
| Mental | | 17.47 (3.6) | 17.46 (3.8) | 17.50 (3.2) | 0.97 | 0.01 |
| MBCB | |  |  |  |  |  |
| Objective burden | | 21.31 (3.3) | 21.38 (3.6) | 21.21 (3.1) | 0.87 | 0.05 |
| Demand burden | | 12.63 (2.2) | 12.59 (1.7) | 12.68 (2.8) | 0.89 | 0.04 |
| Stress burden | | 13.67 (2.3) | 14.03 (1.8) | 13.11 (2.8) | 0.21 | 0.41 |
| Total Score | | 47.6 (5.9) | 48 (5.0) | 47.00 (7.1) | 0.60 | 0.17 |
| PAC | |  |  |  |  |  |
| Self-affirmation | | 19.98 (3.7) | 19.54 (3.4) | 20.63 (4.0) | 0.32 | 0.30 |
| Outlook on life | | 11.91 (2.5) | 11.32 (2.1) | 12.79 (2.9) | 0.06 | 0.59 |
| PAC Total | | 35.51 (5.9) | 34.29 (5.6) | 37.32 (6.1) | 0.09 | 0.51 |
| SD = standard deviation; BCOS = Bakas Caregiving Outcomes Scale; HADS = Hospital Anxiety and Depression Scale; PROMIS = Patient Reported Outcomes Measurement Information System; MBCB = Montgomery Borgatta Caregiver Burden Scale; PAC = Positive Aspects of Caregiving.  ^*^ p-values from t-test or Fisher's exact tests, as appropriate.  ^†^ Effect size: Cohen's d (Cohen, 1988), or d-equivalent (Rosenthal & Rubin, 2003) small: d~0.2, medium d~0.5, large d~0.8. | | | | | | |
